# Supplementary material for: Dose-efficient cryo-electron microscopy for thick samples using tilt- corrected scanning transmission electron microscopy
Source: Nat Methods. 2025 Sep 23;22(10):2138–48. doi: 10.1038/s41592-025-02834-9 (PMC12510875; doi:10.1038/s41592-025-02834-9)
Supplement: Supplementary file 1 — Supplementary Figs. 1–11. [file 41592_2025_2834_MOESM1_ESM.pdf]

nature methods

Supplementary information

https://doi.org/10.1038/s41592-025-02834-9

Dose-efficient cryo-electron microscopy for thick samples using tilt- corrected scanning transmission electron microscopy

In the format provided by the authors and unedited

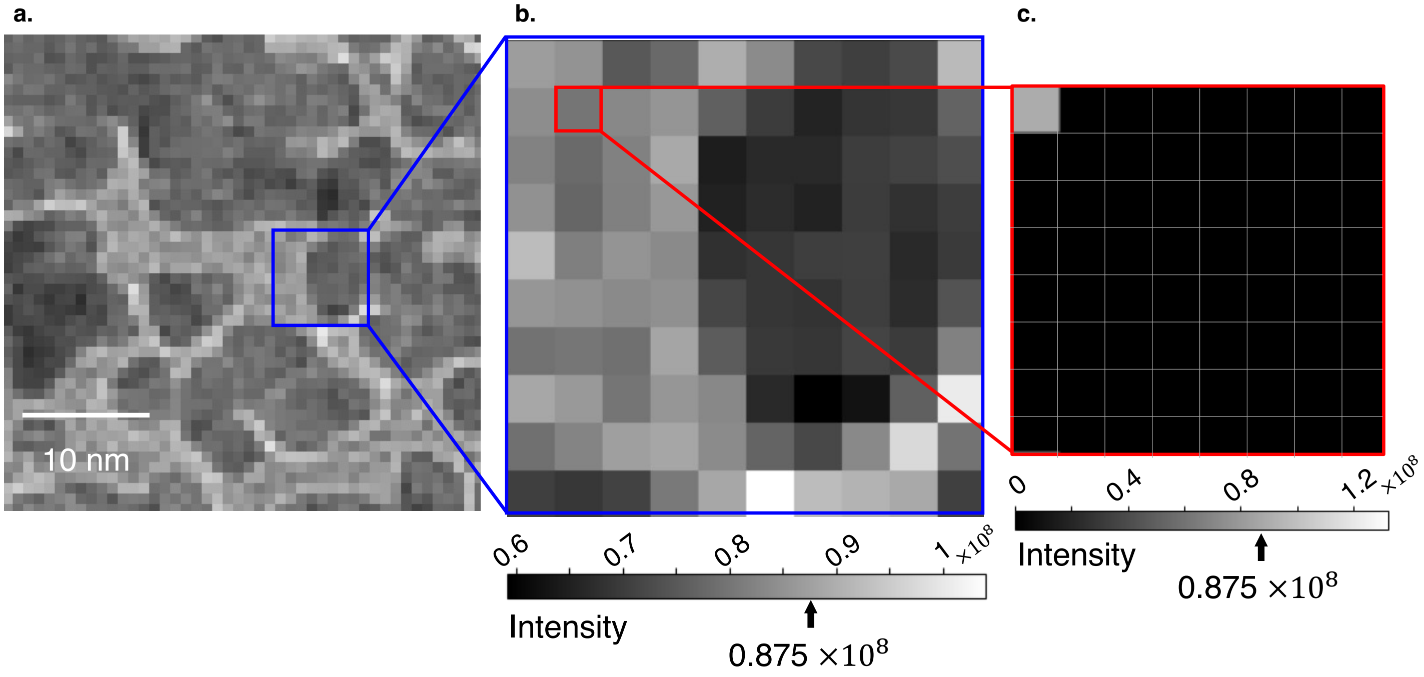

Fig. S1 | To facilitate sub-scan-pixel image shifting, each real-space image formed by a single detector pixel is first padded with zero-intensity pixels. In (a), there is a bright field image formed using a single detector pixel for a standard gold-on-carbon sample under the previously described imaging condition. Each pixel (b) in the image (a) becomes an 8x8 pixel block (c) after padding. The padding process only involves inserting zero values between the original pixels without altering their values.

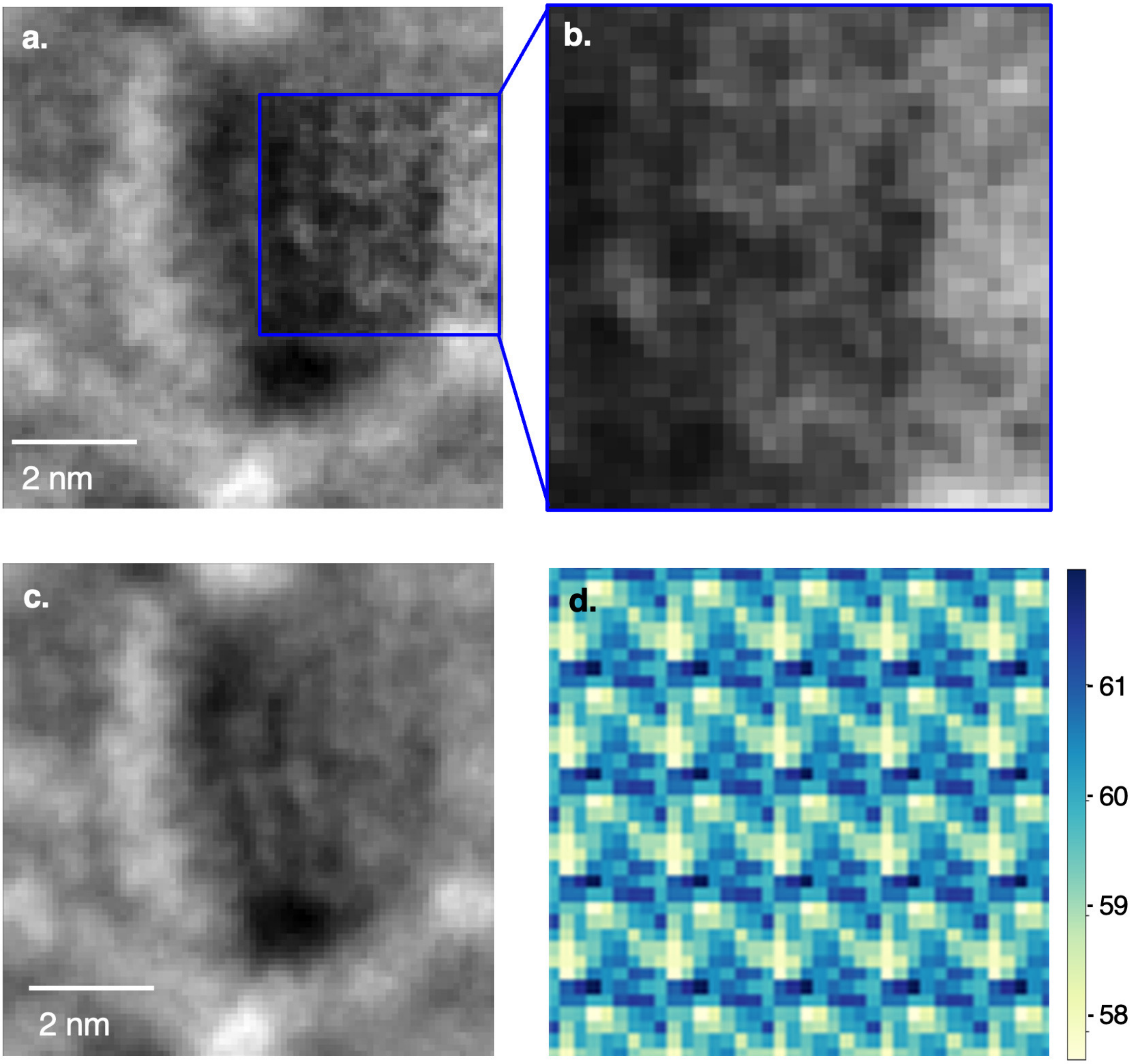

Fig. S2 | Normalization of uneven distributions of sub-pixel shifts: the same up-sampled image shown in Fig. 2d with the periodic intensity variations amplified in the blue-boxed area (b) for better visibility. By tracking the sub-pixel shift distribution (d) and applying an intensity normalization based on this sampling distribution, the periodic artifacts can be corrected.

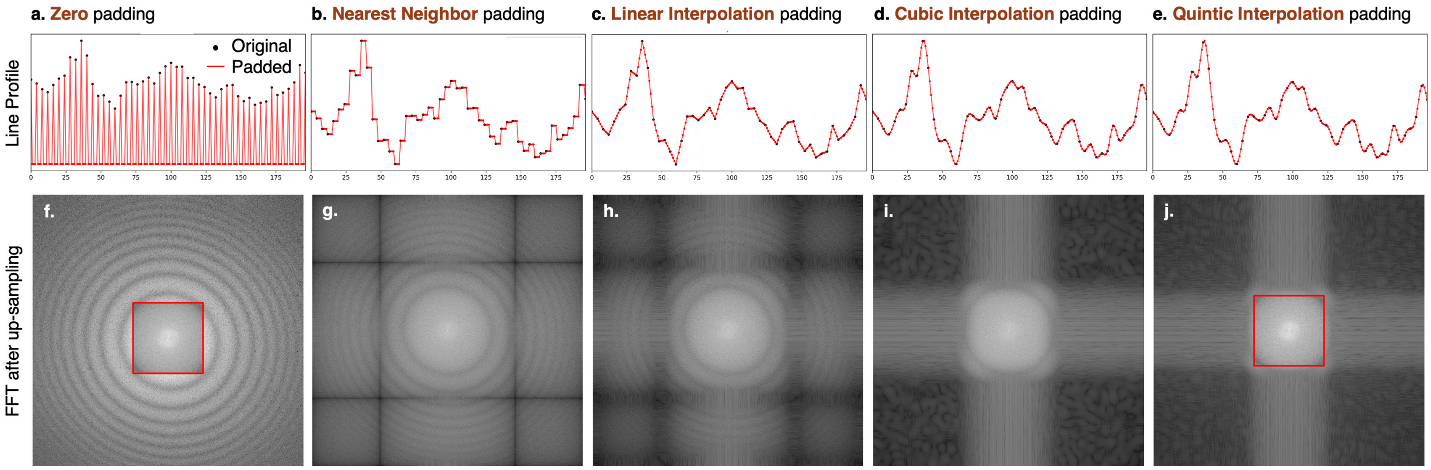

Fig. S3 | Higher-order interpolation padding: line profiles (left) illustrate in 1D the results of padding with zero-intensity pixels and progressing to quintic interpolation values. The corresponding FFTs of the up-sampled images are shown on the right.

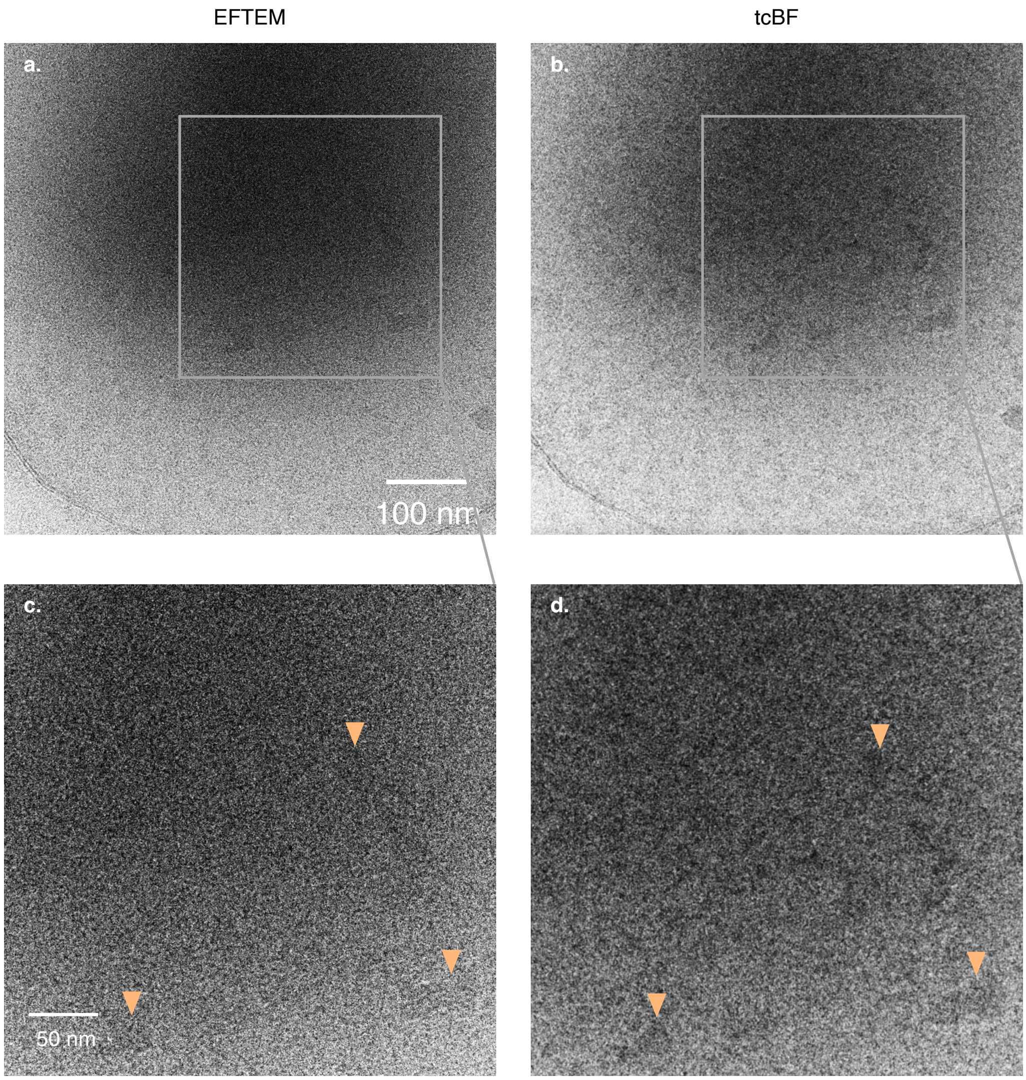

Fig. S4 | Comparisons of EFTEM and tcBF-STEM with different doses, defoci and acquisition orders. Vesicles imaged in (e) EFTEM and (f) tcBF with similar defoci (2.85 and 2.83 μm respectively) . With tcBF (h), features in the interior region of the cell (orange arrows) are more visible than in EFTEM (g). For each comparison, the total dose measured over vacuum and the electron acceleration (300 kV) are the same, and the average thickness was estimated with the EFTEM images using the ratio of I_0/I and the inelastic MFP, similar to Fig. S1. Dose is 14 $e^-/Å^2$ for both image sets. For EFTEM images, slit widths are all 10 eV and defoci are measured with CTFFIND4[43]. For tcBF images, defoci are measured with the image shifts. Defoci, thickness and other experimental details in Table I.

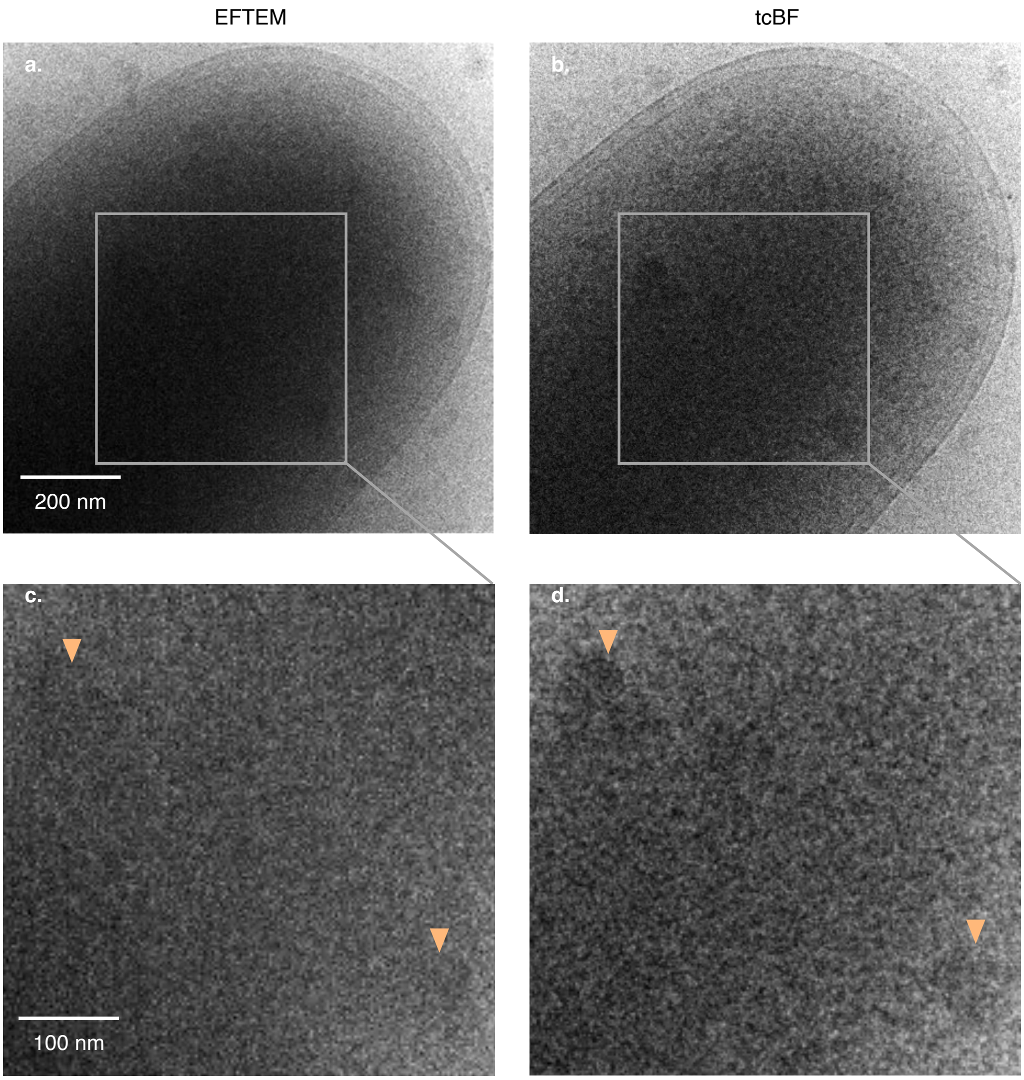

Fig. S5 | Comparisons of EFTEM and tcBF-STEM with different doses, defoci and acquisition orders. Intact *E.coli* cell imaged at low dose (0.5 $e^{-}/Å^{2}$) with (i) EFTEM and (j) tcBF. TcBF (l) is able to resolve features (orange arrows) that are otherwise indiscernible with EFTEM (k).

For each comparison, the total dose measured over vacuum and the electron acceleration (300 kV) are the same, and the average thickness was estimated with the EFTEM images using the ratio of I_0/I and the inelastic MFP, similar to Fig. S1. For EFTEM images, slit widths are all 10 eV and defoci are measured with CTFFIND4[43]. For tcBF images, defoci are measured with the image shifts. Defoci, thickness and other experimental details in Table I.

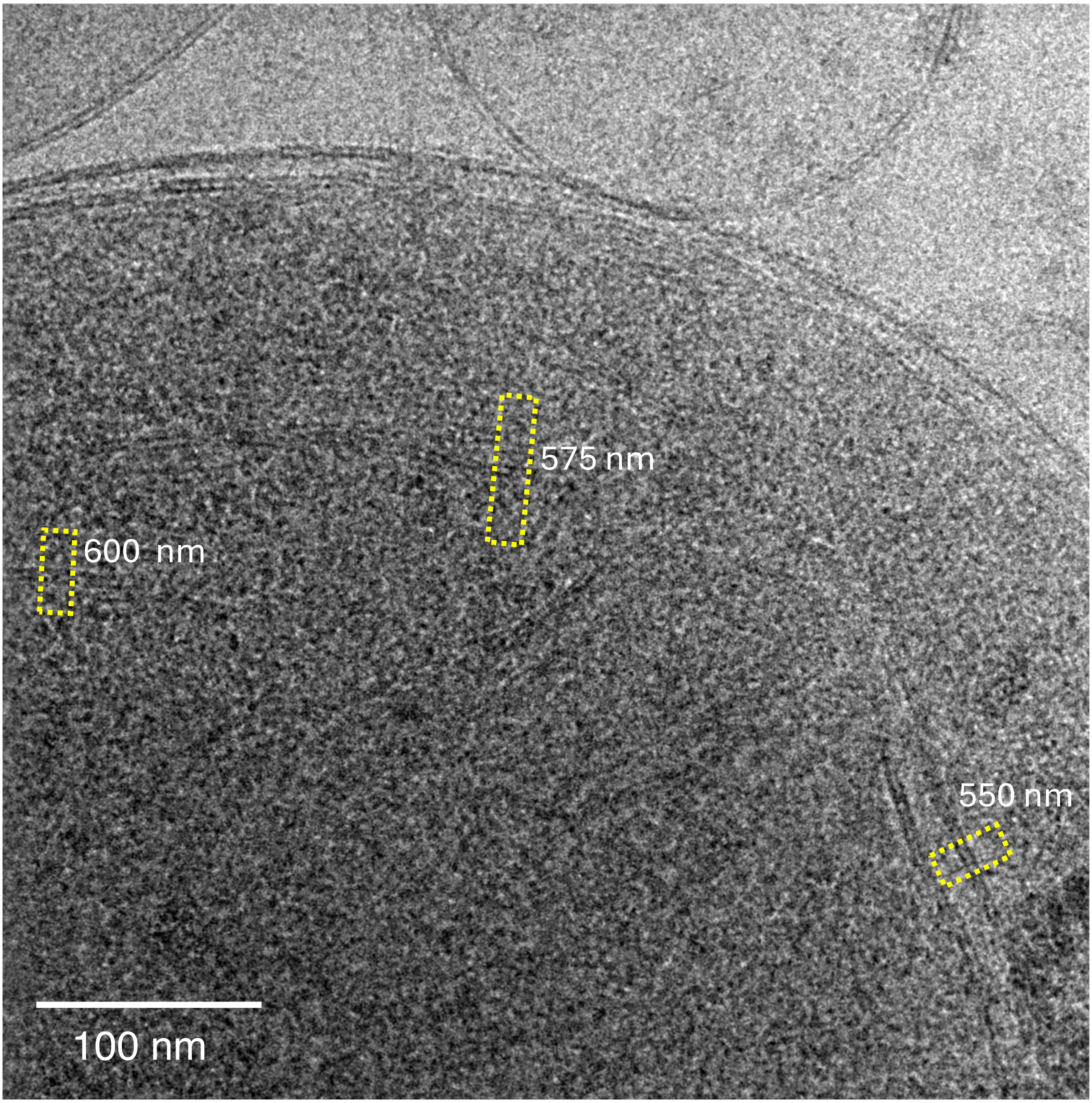

Fig. S6 | Locations from Figure 2x showing the approximate location for the line profiles taken across membranes to measure fringe contrast.

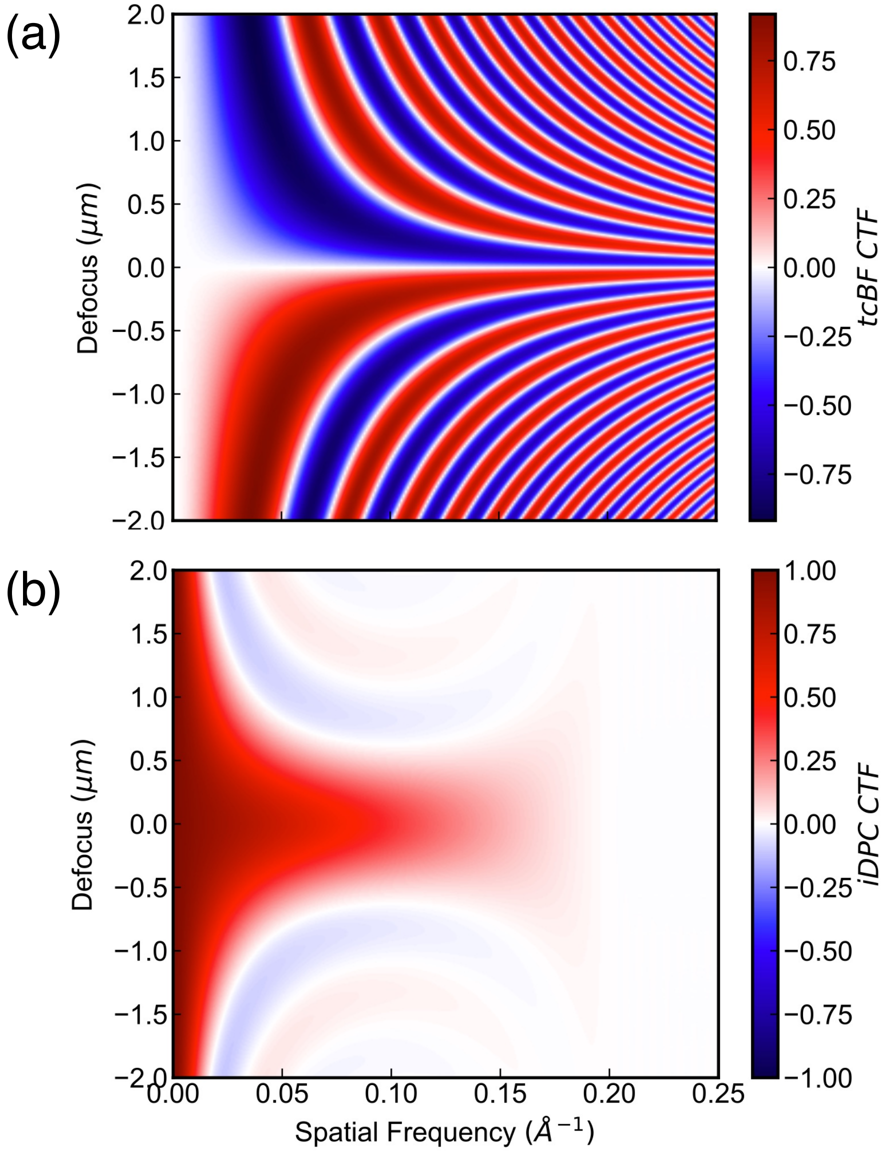

Fig. S7 | Contrast Transfer function versus defocus for tcBF (a) and iDPC (b) respectively. For tcBF, there are rapid contrast reversals at high spatial frequencies as a function of defocus. However, low spatial frequencies are transferred efficiently, and over a wider defocus range. For tcBF a 5.5 mrad probe-forming aperture is chosen, but the precise value affects only the envelope and not the fringe position. b) shows that for iDPC, the optimal defocus is at 0 (i.e. the midpoint of the sample), however the apparently good information transfer at low frequency is misleading, and not reflected in the DQE (see fig 4c and suppl fig x+1). For iDPC a 2 mrad probe-forming aperture is selected to extend the depth-of-focus.

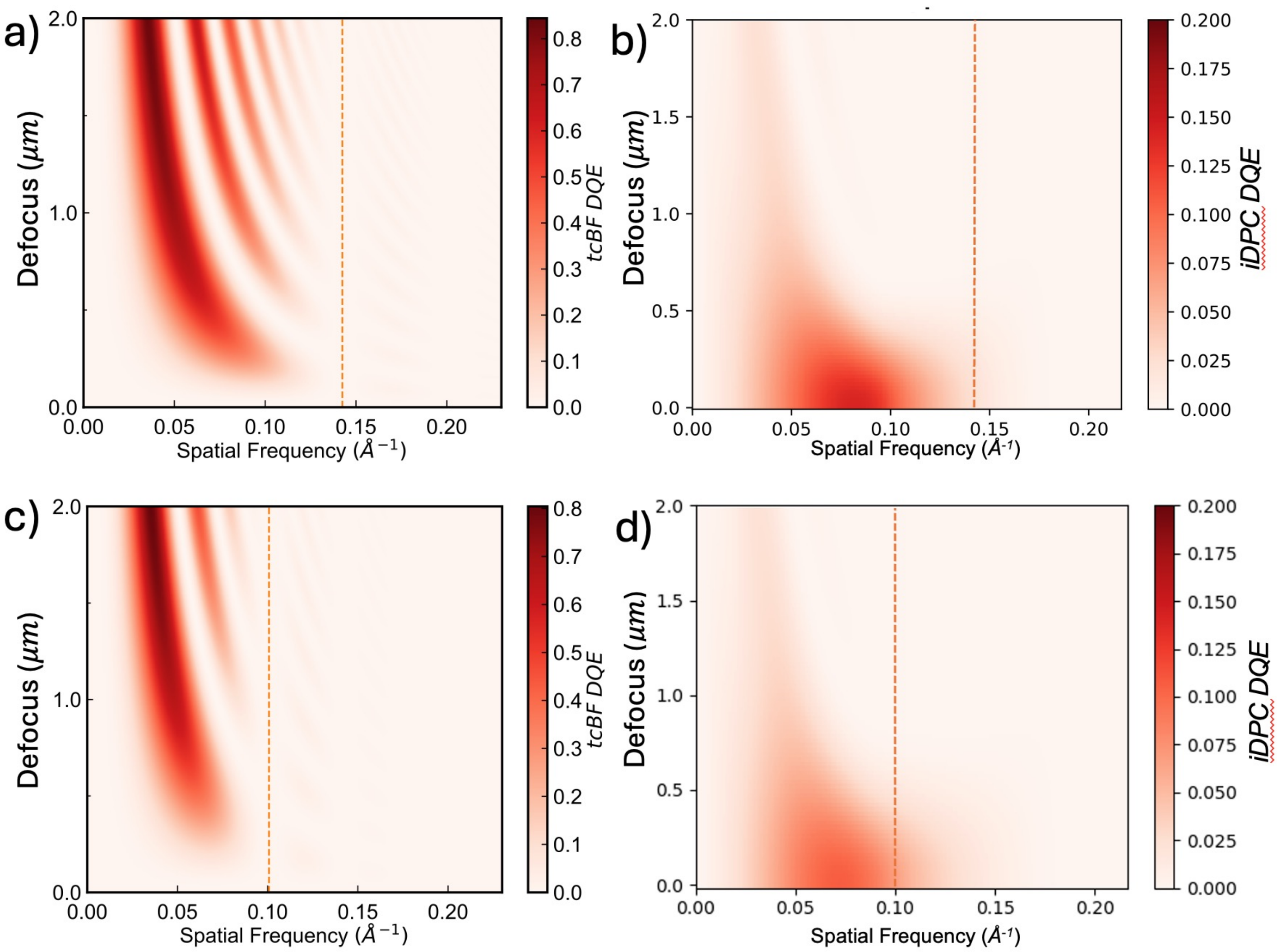

Fig. S8 | Determining the DQE as a function of defocus for a 500-nm thick sample (a,b) and a 1 μm-thick sample (c,d) for tcBF (a,c) and iDPC (b,d) respectively. Averaging over 500 nm approximates the optical limits to image contrast for the projected contrast from a 500 nm-thick sample in the absence of multiple scattering. .The left panels a) and c) show that for tcBF, a wide range of defocus values can be tolerated. The DQE peaks at slightly above 0.8, but there are multiple zeros in the CTF, as in conventional TEM. For tcBF a 5.5 mrad probe-forming aperture is chosen, but the precise value affects only the envelope and not the fringe position. b) and d) shows that for iDPC, the optimal defocus is at 0 (i.e. the midpoint of the sample). For iDPC a 2 mrad probe-forming aperture is selected to extend the depth-of-focus (and larger apertures will have a decreased DQE and a 5.5 mrad aperture for iDPC would have a too-short depth of focus to be clearly visible at this scale plot). Note that the maximum DQE for iDPC is 0.2 compared to 0.8 for tcBF. The orange dashed lines show the expected maximum spatial frequency, $d = \sqrt{0.5\lambda\,\Delta z}$, that can be transferred for a defocus spread Δz.

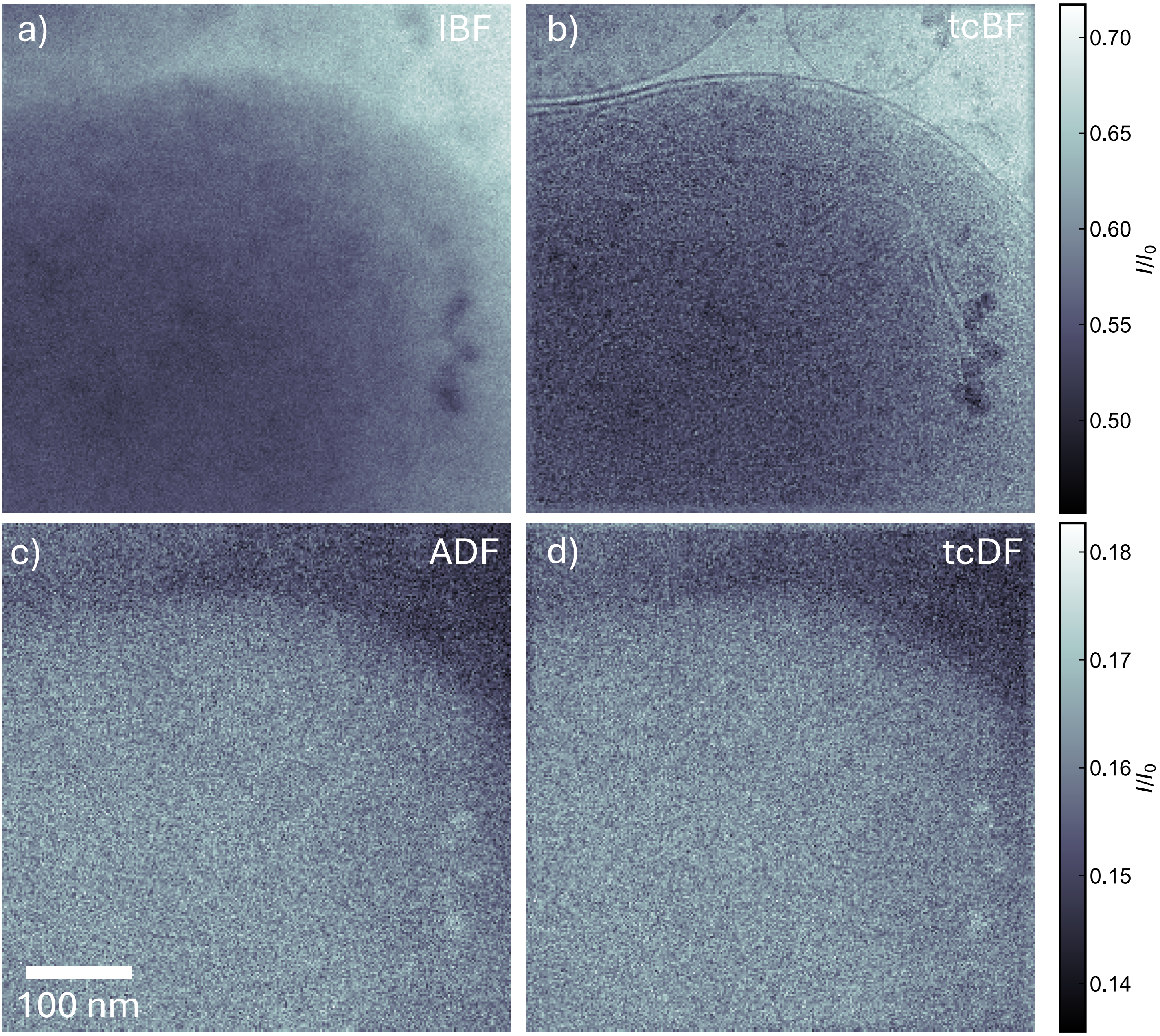

Fig. S9 | Comparison of different imaging modes for the data set from main figure 3: a) Incoherent Bright field (IBF); b) tilt-correct bright field (tcBF); c) annular dark field (ADF); d) tilt-corrected dark field, all without upsampling, and at 2-micron defocus. At this defocus, the tcBF images retains sharp contrast, and comprise 50-70% of the incident beam. The tcDF formed from electrons scattered outside the aperture is more blurred, as are the IBF and ADF incoherent images.

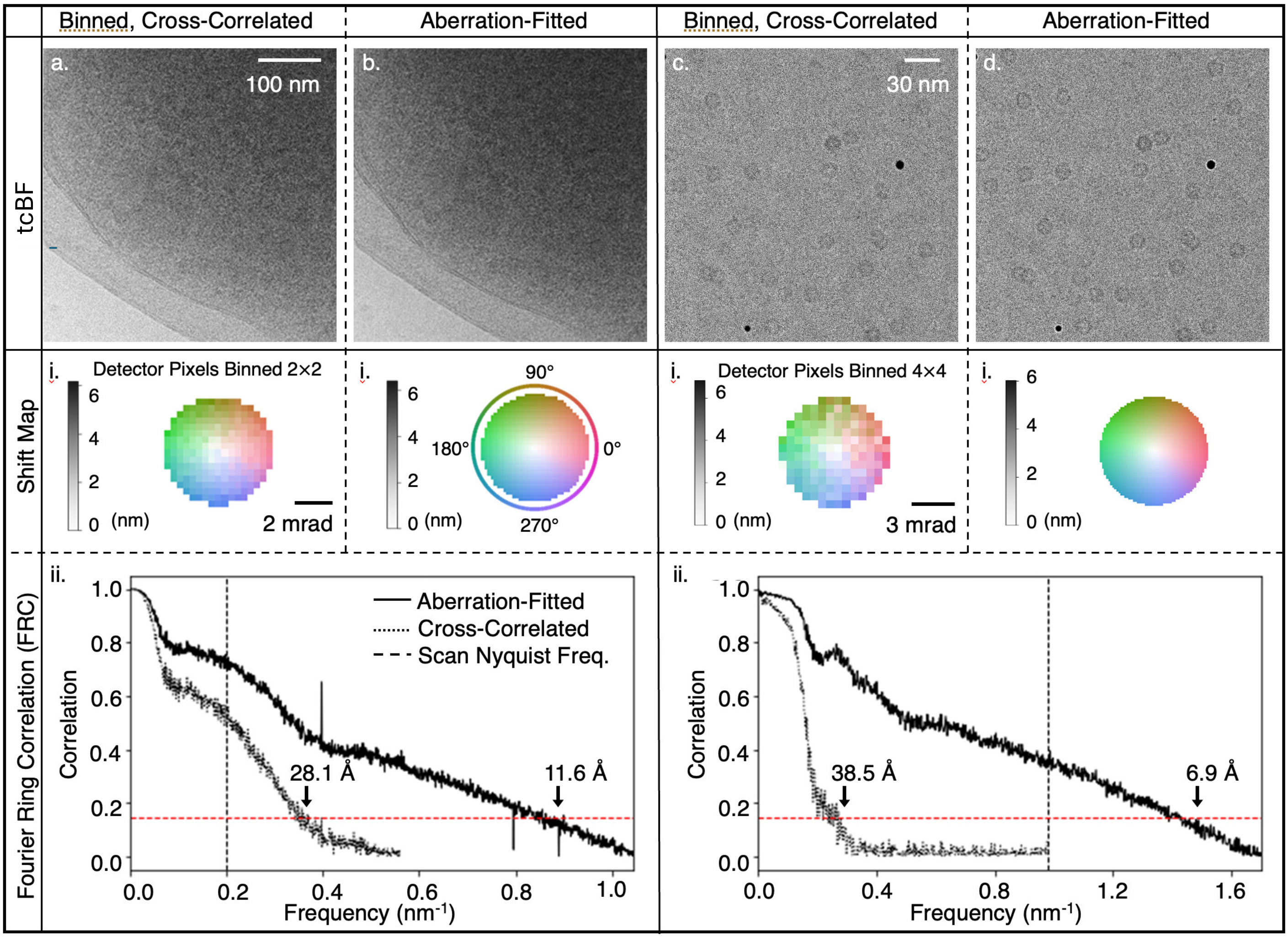

Fig. S10 | To overcome the low-SNR challenge for imaging frozen-hydrated samples, 4-by-4 detector pixels are combined for successful cross-correlation. (a) is a thick E.coli sample. Fitting the shifts to the aberration function and applying the results to the original detector pixels help restore the information from individual detector pixels and improve image shift accuracy (b) and reveals the bilayer cell membrane and details in the interior of the cell. The maps in the insets (i) present the shifts of images formed by each detector pixel, with the intensities indicating the magnitudes and the colors corresponding to the directions. The Fourier Ring Correlating (FRC) in the inset (ii) confirms the resolution enhancement by leveraging aberration, improving the cut-off resolution from 28.1 Å to 11.6 Å . For a thinner apoferritin sample, (c) is the tcBF image with image shifts resolved on binned detector pixels. Leveraging aberration fitting results (d) pushes the resolution from 38.5 Å to 6.9 Å (inset (ii)). The 1/7 correlation threshold and the Nyquist sampling limit are labelled in the FRC plot. The images in (a) to (d) are cropped to show and the FRCs are computed with the full field of view. To calculate the FRC for tcBF-STEM, we generate two tcBF-STEM images from a single dataset by choosing alternating pixels within the BF disks and then reconstructing each subset independently. The normalized cross-correlation coefficient between the two resulting images represents the FRC of the dataset.

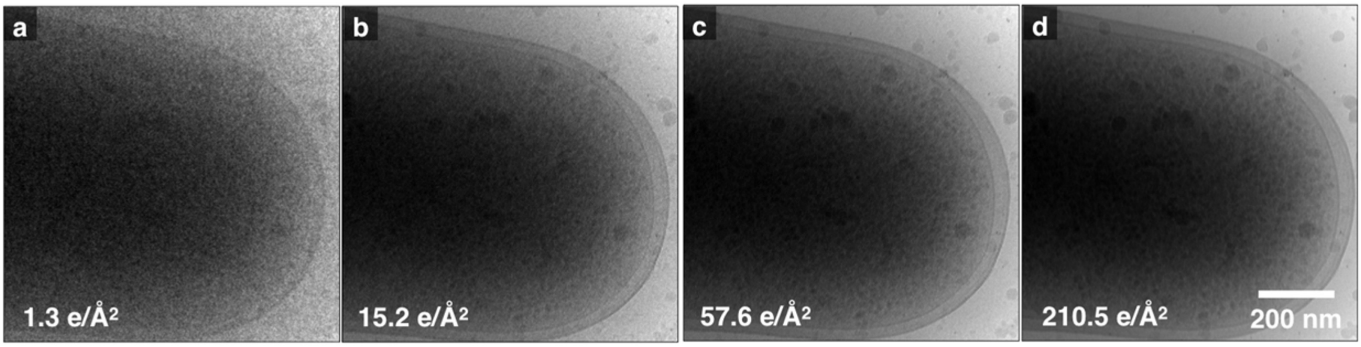

Fig.S11 | Dose Tolerance for tcBF-STEM images collected sequentially with increasing dose: (a) 1.3 $e^-/Å^2$, (b) 15.2 $e^-/Å^2$, (c) 57.6 $e^-/Å^2$, (d) 210.5 $e^-/Å^2$. Large-length-scale features in the specimen appear tolerant to a high cumulative dose, with no bubbling appearing even in the final exposure where the cumulative dose is 286 $e^-/Å^2$.
