# Supplementary material for: Dose-efficient cryo-electron microscopy for thick samples using tilt- corrected scanning transmission electron microscopy
Source: Nat Methods. 2025 Sep 23;22(10):2138–48. doi: 10.1038/s41592-025-02834-9 (PMC12510875; doi:10.1038/s41592-025-02834-9)
Supplement: Supplementary file 2 — Reporting Summary [file 41592_2025_2834_MOESM2_ESM.pdf]

nature portfolio

Corresponding author(s): Yue Yu

Last updated by author(s): May 4, 2025

Reporting Summary

Nature Portfolio wishes to improve the reproducibility of the work that we publish. This form provides structure for consistency and transparency in reporting. For further information on Nature Portfolio policies, see our Editorial Policies and the Editorial Policy Checklist.

Statistics

For all statistical analyses, confirm that the following items are present in the figure legend, table legend, main text, or Methods section.

n/a	Confirmed	
☐	☒	The exact sample size (*n*) for each experimental group/condition, given as a discrete number and unit of measurement
☐	☒	A statement on whether measurements were taken from distinct samples or whether the same sample was measured repeatedly
☒	☐	The statistical test(s) used AND whether they are one- or two-sided<br>*Only common tests should be described solely by name; describe more complex techniques in the Methods section.*
☐	☒	A description of all covariates tested
☐	☒	A description of any assumptions or corrections, such as tests of normality and adjustment for multiple comparisons
☐	☒	A full description of the statistical parameters including central tendency (e.g. means) or other basic estimates (e.g. regression coefficient) AND variation (e.g. standard deviation) or associated estimates of uncertainty (e.g. confidence intervals)
☒	☐	For null hypothesis testing, the test statistic (e.g. *F*, *t*, *r*) with confidence intervals, effect sizes, degrees of freedom and *P* value noted<br>*Give P values as exact values whenever suitable.*
☒	☐	For Bayesian analysis, information on the choice of priors and Markov chain Monte Carlo settings
☒	☐	For hierarchical and complex designs, identification of the appropriate level for tests and full reporting of outcomes
☒	☐	Estimates of effect sizes (e.g. Cohen's *d*, Pearson's *r*), indicating how they were calculated

Our web collection on statistics for biologists contains articles on many of the points above.

Software and code

Policy information about availability of computer code

Data collection	EMPAD GUI 1.2.2 (2021-08-20) for 4d-stem data in figure 3 and fig 4 and supplementary fig s4 and fig s6.
Data analysis	Python packages for tcBF-STEM are available on GitHub at https://github.com/yyu2017/tcBFSTEM. Additionally,example data and notebook are accessible on Code Ocean DOI 10.24433/CO.4000019.v1. The code for analyzing contrast and signal-to-noise are available as source data (ED_fig8_plotlineprofiles.ipynb) Cryosparc version 4.6.

For manuscripts utilizing custom algorithms or software that are central to the research but not yet described in published literature, software must be made available to editors and reviewers. We strongly encourage code deposition in a community repository (e.g. GitHub). See the Nature Portfolio guidelines for submitting code & software for further information.

Data

Policy information about availability of data

All manuscripts must include a data availability statement. This statement should provide the following information, where applicable:

- Accession codes, unique identifiers, or web links for publicly available datasets
- A description of any restrictions on data availability
- For clinical datasets or third party data, please ensure that the statement adheres to our policy

The 4D-STEM datasets for Figures 3, Ext. Fig 7, Suppl. Fig. S4, along with the corresponding EFTEM images acquired in the same regions, are publicly accessible on Zenodo55. The primary map, half maps, FSC, and mask used for the reported FSC in Fig.4 have been deposited on Electron Microscopy Data Bank (EMDB) under ID

EMD-48236. The same for Ext. Data Fig. 9 is deposited under EMDB- 70328. The model docked in the map shown in Fig.4 is available from the Protein Data Bank (PDB) under accession code PDB_1DWN. The raw data of the lineprofiles in Fig.3e-f, and Ext. Data Fig.8, are provided in the source data.

Research involving human participants, their data, or biological material

Policy information about studies with human participants or human data. See also policy information about sex, gender (identity/presentation), and sexual orientation and race, ethnicity and racism.

Reporting on sex and gender	N/A
Reporting on race, ethnicity, or other socially relevant groupings	N/A
Population characteristics	N/A
Recruitment	N/A
Ethics oversight	N/A

Note that full information on the approval of the study protocol must also be provided in the manuscript.

Field-specific reporting

Please select the one below that is the best fit for your research. If you are not sure, read the appropriate sections before making your selection.

☒ Life sciences ☐ Behavioural & social sciences ☐ Ecological, evolutionary & environmental sciences

For a reference copy of the document with all sections, see nature.com/documents/nr-reporting-summary-flat.pdf

Life sciences study design

All studies must disclose on these points even when the disclosure is negative.

Sample size	No statistical methods were used to predetermine sample size. The sample sizes were chosen to cover a range of sample thicknesses, acquisition orders, and other relevant parameters, as defoci et al. The consistency in trends observed across these comparisons was deemed sufficient to consider the sample size adequate for the study's objectives.
Data exclusions	No data were excluded.
Replication	The trend of observing higher collection efficiency of tcBF-STEM compared to EFTEM was successfully replicated across all five comparisons.
Randomization	Randomization was not applied in this study as the selection of sample areas for method comparison was based on thickness. The same sample areas were imaged by both tcBF-STEM and EFTEM methods, making randomization unnecessary for this comparison.
Blinding	Blinding was not applicable in this study because the same sample areas were imaged by both tcBF-STEM and EFTEM methods, making it unnecessary to conceal the group assignments from the researchers or analysts.

Reporting for specific materials, systems and methods

We require information from authors about some types of materials, experimental systems and methods used in many studies. Here, indicate whether each material, system or method listed is relevant to your study. If you are not sure if a list item applies to your research, read the appropriate section before selecting a response.

Materials & experimental systems

n/a	Involved in the study	
☒	☐	Antibodies
☐	☒	Eukaryotic cell lines
☒	☐	Palaeontology and archaeology
☒	☐	Animals and other organisms
☒	☐	Clinical data
☒	☐	Dual use research of concern
☒	☐	Plants

Methods

n/a	Involved in the study	
☒	☐	ChIP-seq
☒	☐	Flow cytometry
☒	☐	MRI-based neuroimaging

Eukaryotic cell lines

Policy information about cell lines and Sex and Gender in Research

Cell line source(s)	HEK293T cells
Authentication	None of the cell lines were authenticated.
Mycoplasma contamination	Cell lines were not tested for mycoplasma contamination.
Commonly misidentified lines (See ICLAC register)	N/A

Plants

Seed stocks	N/A
Novel plant genotypes	N/A
Authentication	N/A
